# Using Reinforcement Learning to Provide Stable Brain-Machine Interface Control Despite Neural Input Reorganization

**DOI:** 10.1371/journal.pone.0087253

**Published:** 2014-01-30

**Authors:** Eric A. Pohlmeyer, Babak Mahmoudi, Shijia Geng, Noeline W. Prins, Justin C. Sanchez

**Affiliations:** 1 Department of Biomedical Engineering, University of Miami, Coral Gables, Florida, United States of America; 2 Department of Neurosurgery, Emory University, Atlanta, Georgia, United States of America; 3 Department of Neuroscience, University of Miami, Miami, Florida, United States of America; 4 Miami Project to Cure Paralysis, University of Miami, Miami, Florida, United States of America; Georgia State University, United States of America

## Abstract

Brain-machine interface (BMI) systems give users direct neural control of robotic, communication, or functional electrical stimulation systems. As BMI systems begin transitioning from laboratory settings into activities of daily living, an important goal is to develop neural decoding algorithms that can be calibrated with a minimal burden on the user, provide stable control for long periods of time, and can be responsive to fluctuations in the decoder’s neural input space (e.g. neurons appearing or being lost amongst electrode recordings). These are significant challenges for static neural decoding algorithms that assume stationary input/output relationships. Here we use an actor-critic reinforcement learning architecture to provide an adaptive BMI controller that can successfully adapt to dramatic neural reorganizations, can maintain its performance over long time periods, and which does not require the user to produce specific kinetic or kinematic activities to calibrate the BMI. Two marmoset monkeys used the Reinforcement Learning BMI (RLBMI) to successfully control a robotic arm during a two-target reaching task. The RLBMI was initialized using random initial conditions, and it quickly learned to control the robot from brain states using only a binary evaluative feedback regarding whether previously chosen robot actions were good or bad. The RLBMI was able to maintain control over the system throughout sessions spanning multiple weeks. Furthermore, the RLBMI was able to quickly adapt and maintain control of the robot despite dramatic perturbations to the neural inputs, including a series of tests in which the neuron input space was deliberately halved or doubled.

## Introduction

Brain-machine interface (BMI) research has made significant advances in enabling human subjects to control computer and robotic systems directly from their neural activity [1]–[6]. These achievements have been supported by neural decoding studies that have shown how functional mappings can be made between single neuron activity, local field potentials (LFPs), and electrocorticograms (ECoG) and kinematics, kinetics, and muscle activation [7]–[19]. Such research has revealed multiple factors that influence neural decoding accuracy on even short timescales (hours to days). For example, performance can be enhanced or degraded by the quantity, type and stability of the neural signals acquired [7], [10], [11], [13], [14], [16], [18], [19], the effects of learning and plasticity [8], [10], [17], [18], availability of physical signals for training the neural decoders [2], [3], [8], and duration of decoder use [7], [12], [17]. These conditions create a dynamic substrate from which BMI designers and users need to produce stable and robust BMI performance if the systems are to be used for activities of daily living and increase independence for the BMI users.

Two of the particularly significant challenges to BMI neural decoders include how to create a decoder when a user is unable to produce a measureable physical output to map to the neural activity for training the decoder, and how to maintain performance over both long and short timescales when neural perturbations (inevitably) occur. For BMIs that use chronically implanted microelectrode arrays in the brain, these perturbations include the loss or addition of neurons to the electrode recordings, failure of the electrodes themselves, and changes in neuron behavior that affect the statistics of the BMI input firing patterns over time.

The first challenge includes situations such as paralysis or limb amputation, in which there is no explicit user-generated kinematic output available to directly create a neural decoder. To address this, some studies have utilized carefully structured training paradigms that use desired target information and/or imagined movements to calibrate the BMI controller [1]–[6], [20]–[23]. Other methods involve initializing the decoder with values based on baseline neural activity, ipsilateral arm movements, or using randomized past decoder parameters, and then refining the decoder [23]. These methods all involve using supervised learning methods to adapt the decoder to the user’s neural activity until effective BMI control has been achieved.

The second challenge involves adaptation. Adaptation of a neural decoder after its initial calibration can lengthen a BMI’s effective lifetime by compensating for gradual changes in the behavior of the BMI inputs. Several studies that used linear discriminant analysis of electroencephalogram (EEG) data, have shown that unsupervised adaptive methods can be used to update model aspects that do not depend on labeled training data [24]–[26]. However, in most cases adaptive BMI systems have relied purely on supervised adaptation. During supervised adaptation, the training data that is used to calculate the decoder is periodically updated using either additional kinematic data [27], recent outputs of the decoder itself (the current decoder being assumed effective enough to adequately infer the user’s desired BMI output) [28]–[32], or inferred kinematics based on known target information as new trials occur [20], [21], [23].

Rather than using supervised adaptation, we are developing a new class of neural decoders based on Reinforcement Learning (RL) [33], [34]. RL is an interactive learning method designed to allow systems to obtain reward by learning to interact with the environment, and which has adaptation built into the algorithm itself using an evaluative scalar feedback signal [35]. As with supervised adaptation methods, these decoders can adapt their parameters to respond to user performance. Unlike supervised adaptation methods, they use a decoding framework that does not rely on known (or inferred) targets or outputs (such as kinematics) as a desired response for training or updating the decoder. Therefore they can be used even when such information is unavailable (as would be the case in highly unstructured BMI environments), or when the output of the current BMI decoder is random (e.g. an uncalibrated BMI system, or when a large change has occurred within the decoder input space), because they use a scalar qualitative feedback as a reinforcement signal to adapt the decoder. Several studies have shown that RL can be used to control basic BMI systems using EEG signals [36], [37] and neuron activity in rats [38], [39]. We have recently introduced a new type of RL neural decoder, based on the theory of Associative reinforcement learning, that combines elements of supervised learning with reinforcement based optimization [34]. In that work, we used motor neuron recordings recorded during arm movements as well as synthetic neural data, generated by a biomimetic computational model, to show how the decoder could be used to solve simulated neuroprosthetic tasks. These tasks involved both multiple targets and required the controller to perform sequences of actions to reach goal targets. The current study extends that work by applying this new RL decoder to a real-time BMI task, and by testing its performance in that task when large numbers of the BMI inputs are lost or gained.

The RL neural decoder was evaluated under three basic conditions: an absence of explicit kinetic or kinematic training signals, large changes (i.e. perturbations) in the neural input space, and control across long time periods. Two marmoset monkeys used the Reinforcement Learning BMI (RLBMI) to control a robot arm during a two-target reaching task. Only two robot actions were used to emphasize the relationship between each specific robot action, the feedback signal, perturbations, and the resulting RLBMI adaptation. The RLBMI parameters were initially seeded using random numbers, with the system only requiring a simple ‘good/bad’ training signal to quickly provide accurate control that could be extended throughout sessions spanning multiple days. Furthermore, the RLBMI automatically adapted and maintained performance despite very large perturbations to the BMI input space. These perturbations included either sudden large-scale losses or additions of neurons amongst the neural recordings.

## Materials and Methods

### Overview

We developed a closed-loop BMI that used an actor-critic RL decoding architecture to allow two (PR and DU) marmoset monkeys (*Callithrix jacchus*) to control a robot arm during a two-choice reaching task. The BMI was highly accurate (∼90%) both when initialized from random decoder initial conditions at the beginning of each experimental session and when tested across a span of days to weeks. We tested the robustness of the decoder by inducing large perturbations (50% loss or gain of neural inputs), and the BMI was able to quickly adapt within 3–5 trials.

### Ethics Statement

All animal care, surgical, and research procedures were performed in accordance with the National Research Council Guide for the Care and Use of Laboratory Animals of the National Institutes of Health. They were approved by the University of Miami Institutional Animal Care and Use Committee (protocol: 10–191). The marmosets are housed in a climate controlled environment, with their overall care closely supervised by the University of Miami Division of Veterinary Services (DVR). The animals are regularly inspected by DVR staff to verify healthy behavior. In addition, the senior veterinary staff performs regular checks that include physical examinations and blood tests. The marmosets receive a daily diet that combines fresh fruits with dry and wet species-specific feeds and have access to water *ad libitum*. The marmosets are given environmental enrichments, which include: toys, novelty food treats, and various privacy houses for play and sleep to ensure animal welfare. Surgical procedures are carried out under sterile conditions in a dedicated operating suite under the supervision of the veterinary staff. Following surgical procedures, lab personnel and DVR staff closely monitor subject health during convalescence until they can be returned to the standard (daily) observation schedule. After the completion of all experiments, the brains are processed for histological evaluation of the recording sites following transcardial perfusion under deep anesthesia.

### Microwire Electrode Array Implantation

Each monkey was implanted with a16-channel tungsten, microelectrode array (Tucker Davis Technologies, Alachua FL) in the motor cortex, targeting arm and hand areas. A craniotomy was opened over the motor area, and the dura resected. The array placement was made using stereotaxic coordinates [40]–[44] and cortical mapping (DU motor implant) using a micropositioner (Kopf Instruments, Tujunga, CA). The implant was secured using anchoring screws, one of which served as reference and ground. The craniotomies were sealed using Genta C∼ment (EMCMBV, Nijmegen, The Netherlands). Surgical anesthesia was maintained using isoflurane (PR) or constant rate ketamine infusion (DU), steroids (dexamethasone) were used to minimize brain edema and swelling, and analgesics (buprenorphine) and antibiotics (cefazolin, cephalexin) were administered postoperatively for 2 and 5 days, respectively.

### Neural Data Acquisition

Neural data were acquired using a Tucker Davis Technologies RZ2 system (Tucker Davis Technologies, Alachua, FL). Each array was re-referenced in real-time using a common average reference (CAR) composed of that particular array’s 16 electrodes (if an electrode failed it was removed from the CAR) to improve SNR [45]. Neural data were sampled at 24.414 kHz and bandpass filtered (300 Hz-5 kHz). Action potential waveforms were discriminated in real-time based on manually defined waveform amplitudes and shapes. The recorded neural data included both multineuron signals and well-isolated single neuron signals (collectively referred to here as neural signals), which were used equivalently in all real-time and offline tests. On average there were 18.3+/−3.1 (mean +/− std) motor neural signals for DU and 21.1+/−0.4 for PR (10 signals for PR following a mechanical connector failure in which half the electrodes were lost). Neural signal firing rates were normalized (between −1 to 1) in real-time by updating an estimate of the neural signals’ maximum firing rates during each experimental trial.

### Actor-critic Reinforcement Learning Brain-machine Interface Control Architecture

**Figure 1:**
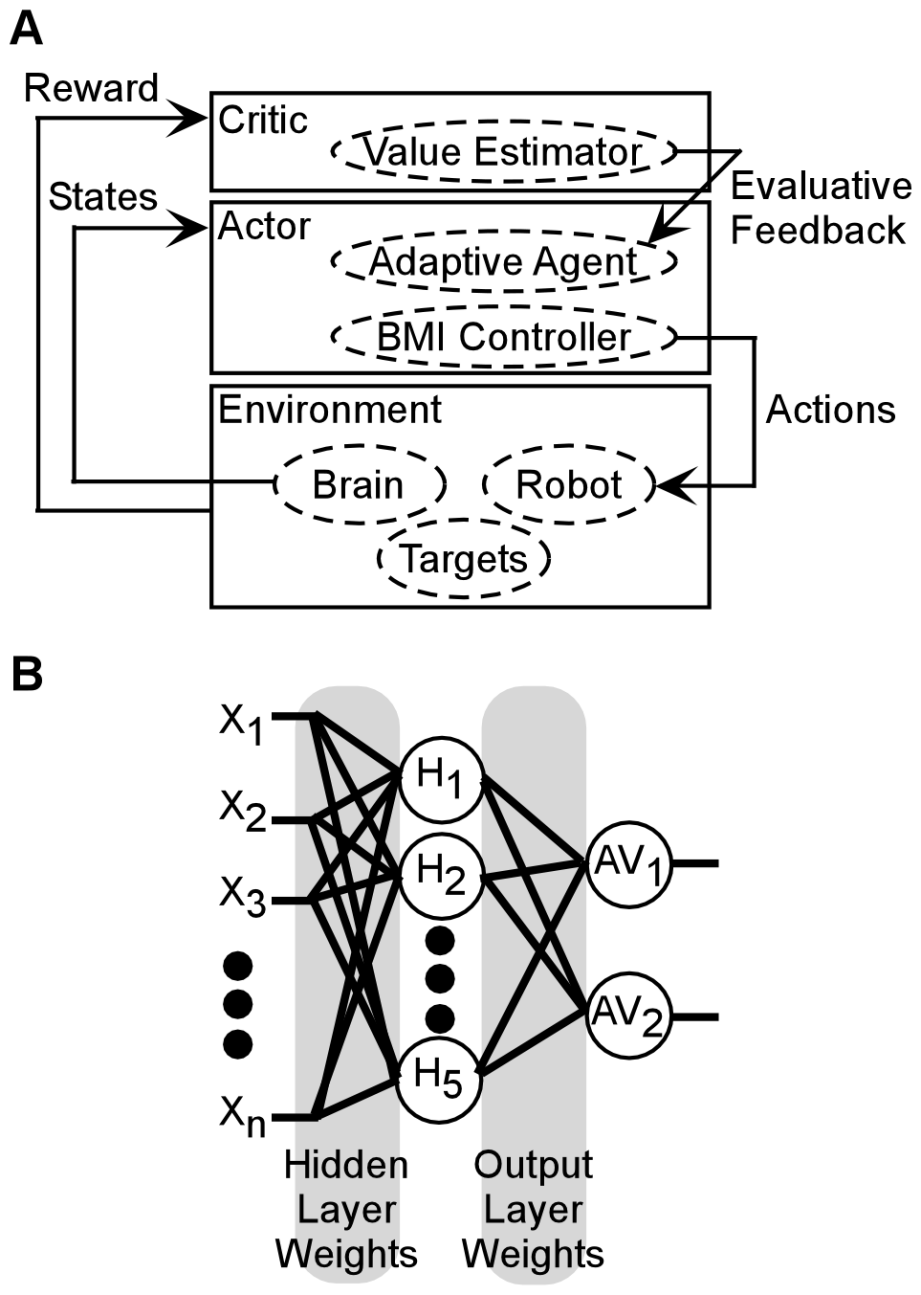
Brain-Machine Interface control architecture using actor-critic reinforcement learning. (A) The architecture’s defining characteristic is the interaction between the actor and critic modules. The actor interacts with the environment by selecting actions given input states (here the BMI Controller). The critic is responsible for producing reward feedback that reflects the actions’ impact on the environment, and which is used by the actor to improve its input to action mapping capability (here the Adaptive Agent). (B) The actor used here is a fully connected three layer feedforward neural network with five hidden (H_i_) and two output (AV_i_) nodes. The actor input (X) was the normalized firing rates of each motor cortex neural signal. Each node was a processing element which calculated spiking probabilities using a tanh function, with the node emitting spikes for positive values.

The actor-critic RLBMI architecture used for these experiments is described in detail in [34]. Briefly, actor-critic systems are characterized by the actor and critic modules, Figure 1A. The actor interacts with the environment by selecting system actions given a specific input state (here neural states). The critic provides an evaluative feedback regarding how successful the actions were in terms of some measure of performance, and which is used to refine the actor’s state to action mapping. The actor was a fully connected 3-layer feedforward neural network, Figure 1B, that used a Hebbian update structure [34]. The actor input (***X***) was a vector (length *n*) of the spike counts for each of the *n* motor cortex neural signals during a two second window following the go cue of each trial. A parsimonious network was chosen for decoding, using only 5 hidden nodes and two output nodes (one for each of the two robot reaching movements). The output of each hidden node (*OutHi*) was a probability of firing (−1 to 1) computed using a hyperbolic tangent function, and in which ***WH_i_*** is the synaptic weights vector between node *i* and the *n* inputs (**b** is a bias term):
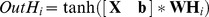
(1)


The output nodes determined the action values (*AV*) for each of the *j* possible robot actions:
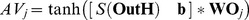
(2)


*S* ***(OutH)*** is a sign function applied to the hidden layer outputs (positive values become +1, negative values become −1), and ***WO_j_*** is the weights matrix between output *j* and the hidden layer. The robot action with the highest action value was implemented each trial. The actor weights were initialized using random numbers, which were updated (Δ**W**) using the critic feedback (*f*):
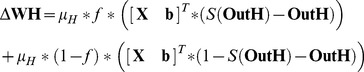
(3)

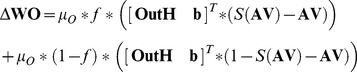
(4)


Feedback is +1 if the previous action selection is rewarded and −1 otherwise. This update equation is composed of two terms that provide a balance between the effects of reward and punishment on the network parameters. Under rewarding conditions, the first term contributes to the changes in the synaptic weights, whereas in the case of punishment both terms will affect the weight update. After convergence to an effective control policy the output of the node tends to the sign function and thus the adaptation will stop automatically [34]. In the current work an ‘ideal’ critic was used that always provided accurate feedback. However, such perfect feedback is not intrinsically assumed by this RL architecture, and there are many potential sources of the feedback in future BMI applications (see **Discussion**). *S()* is again the sign function and µ*_H_* and µ*_O_* are learning rates of the hidden (0.01) and output (0.05) layers, respectively. The update equations are structured so that the local input-output correlation in each node are reinforced using a global evaluative feedback, hence Hebbian reinforcement learning. In the current work, the architecture is applied to a two state problem, but this architecture and update equations 3 and 4 can be directly applied to multistep and multitarget problems, even while still using a binary feedback signal [34].

### Brain-machine Interface Robot Reaching Task

**Figure 2:**
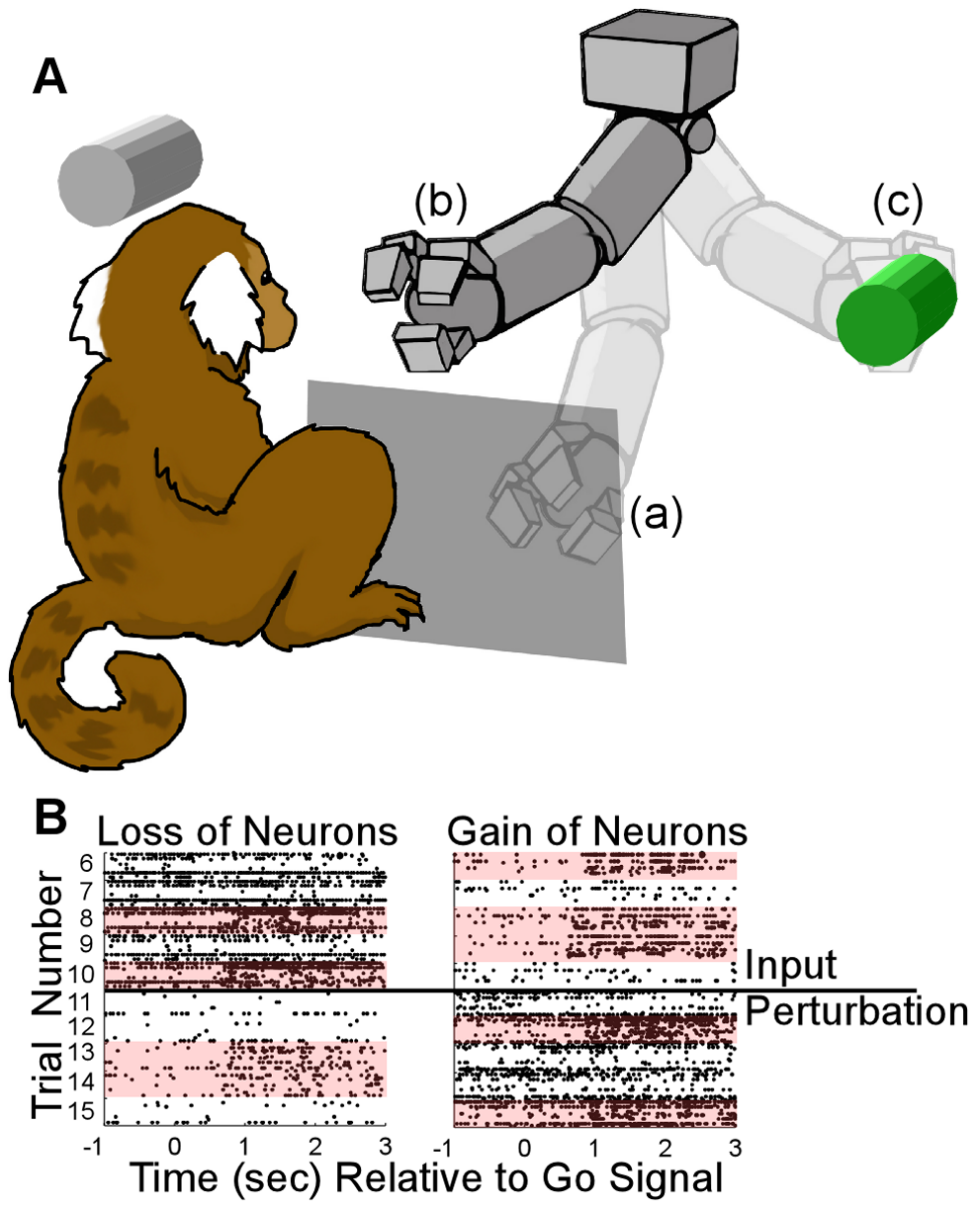
Two target robot reaching task using the RLBMI. The monkeys initiated each trial by placing their hand on a touch sensor for a random hold period. A robot arm then moved out from behind an opaque screen (position a) and presented its gripper to the monkey (position b). A target LED on either monkey’s left (A trials) or right (B trials) was illuminated to indicate the goal reach location. The RLBMI system (Figure 1) used the monkeys’ motor cortex activity to either move the robot to the A or B target (panel A). The monkeys received food rewards only when the RLBMI moved the robot to the illuminated target (position c), Movie S1. Panel B shows examples of the spike rasters for all the neural signals used as inputs to the RLBMI during experiments which tested the effects of neural signals being lost or gained. Data is shown for trials 6–10 (which preceded the input perturbation) and trials 11–15 (which followed the input perturbation). For each trial, all the recorded neural signals are plotted as rows (thus there are multiple rows for a given trial), with data from type A trials being highlighted in red. Differences in firing patterns during the A and B trials are evident both before and after the perturbation, although the RLBMI still had to adapt to compensate for the considerable changes in the overall population activity that resulted from the input perturbations.

The BMI task required the monkeys to make reaching movements with a robot arm to two different spatial locations in order to receive food rewards, Figure 2A and Movie S1. The monkeys initiated trials by placing their hand on a touch sensor for a randomized hold period (700–1200 msec). The hold period was followed by an audio go cue, which coincided with the robot arm moving to the start position. Simultaneously to the robot movement, an LED spatial target on either the monkeys’ left (‘A’ trials) or right (‘B’ trials) was illuminated. Prior to the real-time BMI experiments, the monkeys had been trained to manually control the robot movements by either making or withholding arm movements. During those training sessions, the monkeys moved the robot to the A target by reaching and touching a second sensor, and moved the robot to the B target by keeping their hand motionless on the touchpad, and were rewarded for moving the robot to the illuminated target. The differences in the neuron firing rates shown by the rasters in Figure 2B illustrate how this had trained the monkeys to associate changes in motor activity with moving the robot to the A target, and static motor activity to B target robot movements. In the real-time BMI experiments, the robot movements were determined directly from the monkeys’ motor cortex activity using the actor-critic RL algorithm previously described. A and B trials were presented in a pseudo-random order of roughly equivalent proportions. The monkeys were immediately given food rewards (waxworms/marshmallows) at the end of trials only if they had moved the robot to the illuminated LED target.

In these initial RLBMI tests, we controlled the experiment to examine the basic adaptive capabilities of the RL architecture as a state-based BMI controller, and thus only two robot action states (‘move to target A’ or ‘move to target B’) were used. This allowed us to highlight the relationship between each individual robot action, the feedback training signal, and the resulting adaptive modifications of the RLBMI parameters in a direct and quantifiable manner. This was particularly useful when considering parameter adaptation from wide ranging, random initial conditions, and when we introduced perturbations to the neural input space.

To speed the initial adaptation of the RL algorithm, real time ‘epoching’ of the data was used. After each robot action, the algorithm weights were updated (equations 3 and 4) using not only the most recent trial’s data, but rather with a stored buffer of all the previous trials from that session, with the buffered trials being used to update the weights ten times following each action. The RL was initialized using random numbers and therefore employed random exploratory movements until more effective parameters are learned, thus this epoching helped prevent the monkeys from becoming frustrated at the beginning of sessions by moving the system more rapidly away from purely random actions.

### RLBMI Stability when Initialized from Random Initial Conditions

During real-time closed loop robot control experiments the parameter weights of the RLBMI were initialized with random values, with the RLBMI learning effective action mappings through experience (equations 3 and 4). Performance was quantified as percentage of trials in which the target was achieved. In addition to these closed-loop real-time experiments, we also ran a large number of offline ‘open-loop’ Monte Carlo simulations to exhaustively confirm that the RLBMI was robust in terms of its initial conditions, i.e. that convergence of the actor weights to an effective control state during the real-time experiments had not been dependent on any specific subset of initialization values. For the offline simulations, the neural data and corresponding trial targets for the first 30 trials of several closed-loop BMI sessions from both monkeys (10 sessions for DU and 7 for PR) were used to build a database for open-loop simulations. During the simulations, data from each session were re-run 100 times, and different random initial conditions were used for each test.

### RLBMI Stability during Input Space Perturbations: Loss or Gain of Neuron Recordings

For BMI systems to show truly stable performance, nonstationarities or other changes in the input space should not adversely affect performance. While some changes of the input space can be beneficial, such as neurons changing their firing pattern to better suit the BMI controller [8], [10], [17], [18], [46]–[48], large changes in the firing patterns of the inputs that dramatically remove the input space from that which the BMI had been constructed around are significant problems for BMIs. Such perturbations can result from neurons appearing or disappearing from the electrode recordings, a common occurrence in electrophysiology recordings.

In several closed-loop BMI sessions, we deliberately altered the BMI inputs to test the RLBMI’s ability to cope with large-scale input perturbations. These perturbations were done following the initial learning period so that the RLBMI had already adapted and gained accurate control of the robot prior to the input perturbation. During input loss tests, the real-time spike sorting settings were adjusted (following the 10^th^ trial) so that a random 50% of the neural signals were no longer being detected by the RLBMI. During input gain tests, when the RLBMI was initialized at the beginning of the experiment the real-time spike sorting settings were configured so that the action potentials of a random half of the available neural signals were not being acquired. Then, following the initial adaptation of the RLBMI, the parameters were updated so that the previously avoided signals suddenly appeared as ‘new’ neural signals amongst the BMI inputs.

We verified the real-time input perturbation experimental results with additional offline simulations and during several real-time tests that spanned multiple days. The offline simulation tests used the same Monte Carlo simulation database previously described. For the offline input loss simulation tests, the firing rates for a randomly chosen (during each simulation) half of the neural signals were set to zero after 10 trials and the ability of the RLBMI to compensate was evaluated. Similarly, for the ‘found neuron’ simulations, for each simulation half the inputs were randomly selected to have their firing rates set to zero for the first 10 trials. Finally, during several real-time RLBMI experiments that spanned multiple days, we found that abrupt 50% input losses only caused temporary performance drops even though the system had been adapting for several days prior to the perturbation (see: *RLBMI stability over long time periods* below).

We used the mutual information between the neuron data and the robot task to quantify the impact of input perturbations on the RLBMI input space. The mutual information (MI) between each neural signal (*x*) and the target location (*y*) was determined [49]:
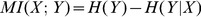
(5)where *H* is the entropy:



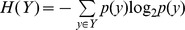
(H(Y) is 1 bit when A and B trials are equally likely) 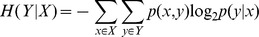


We used Monte Carlo simulations in which different fractions of the neural signals were randomly ‘lost’ (i.e. firing rate became zero) and used the resulting relative change in the average mutual information to gauge the effect of losing neural signal recordings on the RLBMI input space.

### RLBMI Stability Over Long Time Periods

We tested how well the RLBMI would perform when it was applied in closed-loop mode across long time periods. These contiguous multisession tests consisted of a series of robot reaching experiments for each monkey. During the first session, the RLBMI was initialized using a random set of initial conditions. During the follow-up sessions, the RLBMI was initialized from weights that it had learned from the prior session, and then continued to adapt over time (equations 3 and 4).

We also tested the impact of input perturbations during the contiguous multisession experiments. During the contiguous PR tests, a failure in the implant connector resulted in half of the neural signals inputs to the RLBMI being lost. We ran another contiguous session in which the RLBMI successfully adapted to this change to its inputs. This input loss was simulated in two of the contiguous sessions with monkey DU. In those experiments, a random half of the motor neural signals were selected (the same signals in each test), and in those perturbation experiments the firing rates of the selected inputs were set to zero. For comparison purposes, in monkey DU two final contiguous session experiments were also run in which the whole input space remained available to the RLBMI system.

## Results

### Actor-Critic Reinforcement Learning Brain-Machine Interface (RLBMI) Control of Robot Arm

**Figure 3:**
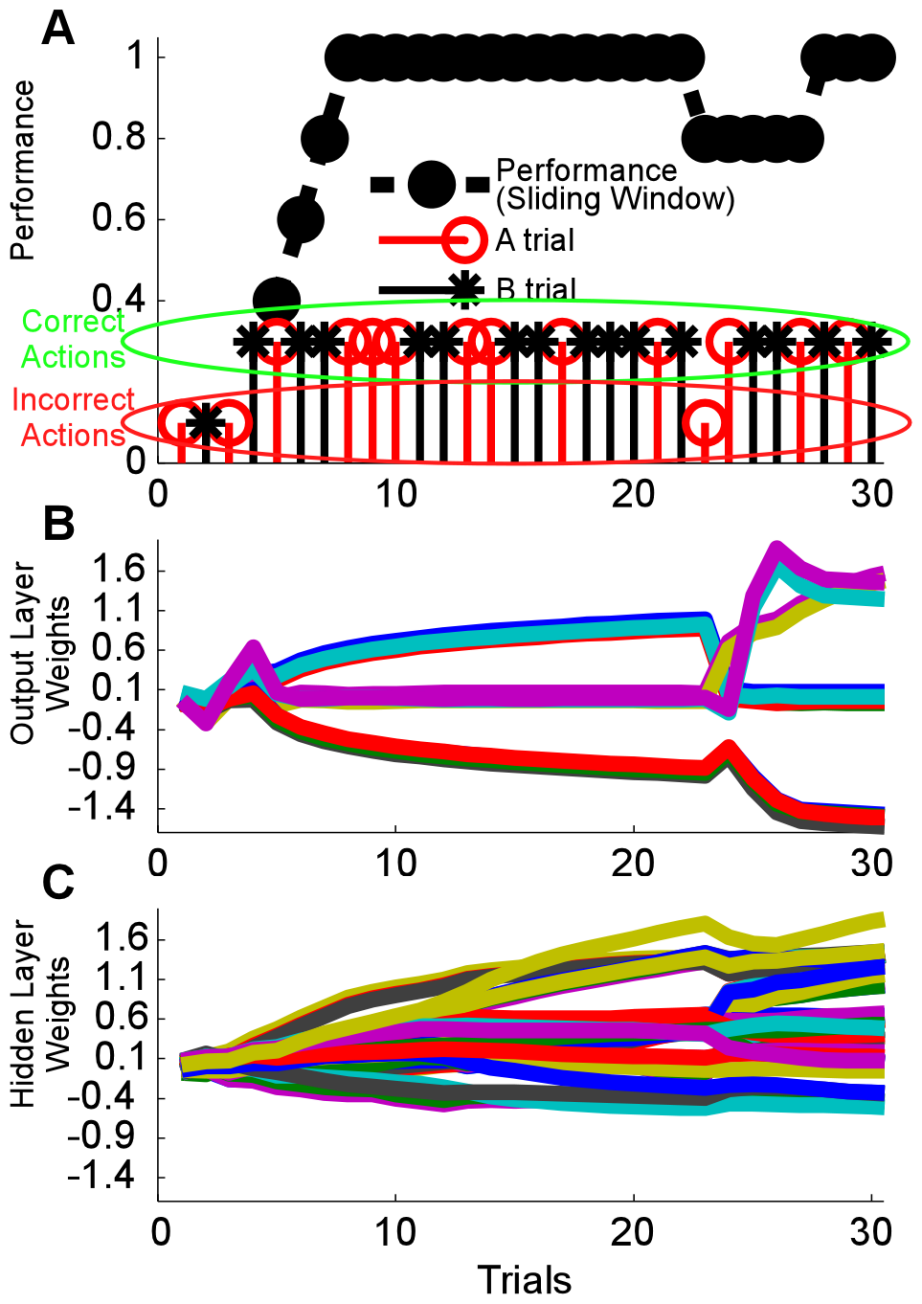
The RLBMI accurately learned to control the robot during closed loop BMI experiments. (A): stems indicate the sequence of the different trials types (O = A trials, * = B trials) with the stem height indicating whether the robot moved to the correct target (taller stem) or not (shorter stem). The dashed line gives the corresponding accuracy of the RLBMI performance within a five trial sliding window. (B and C) show how throughout every trial the RLBMI system gradually adapted each of the individual weights that connected the hidden layer to the outputs (B) as well as all the weights of the connections between the inputs and the hidden layer (C), as the RLBMI learned to control the robot. The shape of these weight trajectories indicate that the system had arrived at a consistent mapping by the fifth trial: at that point the weight adaptation progresses at a smooth rate and the robot is being moved effectively to the correct targets. At trial 23 an improper robot movement resulted in the weights being quickly adjusted to a modified, but still effective, mapping.

The actor-critic RLBMI effectively controlled the robot reaching movements. Figure 3 shows a typical closed loop RLBMI experimental session (PR). Figure 3A shows that the algorithm converged to an effective control state in less than 5 trials, after which the robot consistently made successful movements. The algorithm was initialized using small random numbers (between +/−.075) for the parameter weights (equations 1 and 2). Figure 3B shows the gradual adaptation of the weight values of the two output nodes (equation 4) as the algorithm learned to map neural states to robot actions (Figure 3C shows a similar adaptation progression for the hidden layer weights). The weights initially changed rapidly as the system moved away from random explorations, followed by smooth adaptation and stabilization when critic feedback consistently indicated good performance. Larger adaptations occurred when the feedback indicated an error had been made.

**Figure 4:**
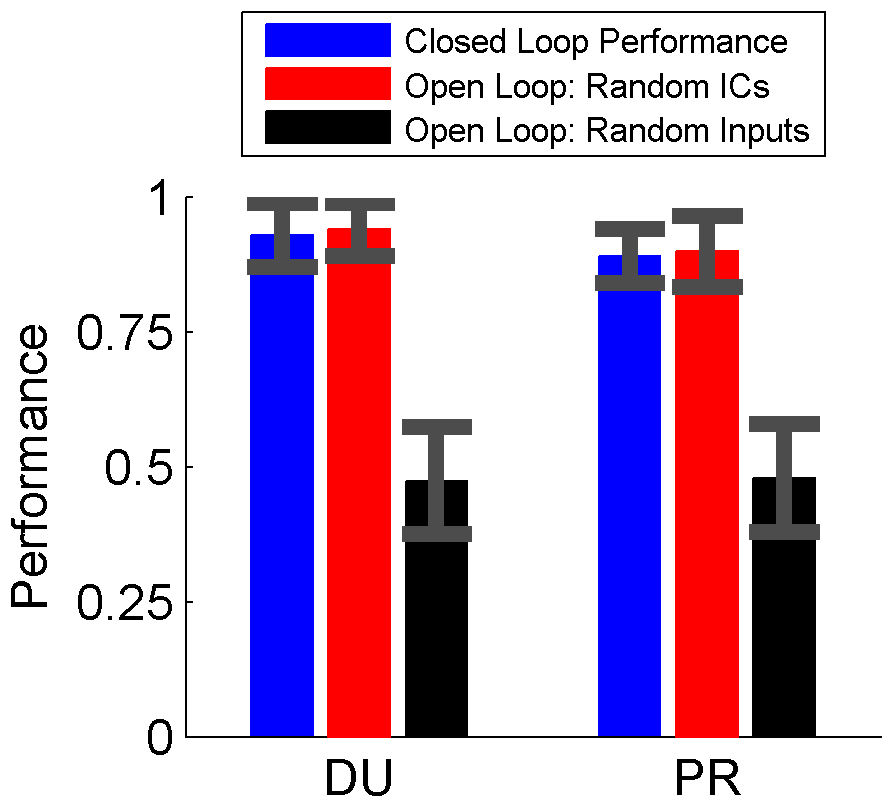
The RLBMI decoder accurately controlled the robot arm for both monkeys. Shown is the accuracy of the decoder (mean +/− standard deviation) following the initial adaptation period (trials 6∶30). Both monkeys had good control during closed loop sessions (blue, DU: 93%, PR: 89%). The open loop simulations (red) confirmed that system performance did not depend on the initial conditions (ICs) of the algorithm weight parameters (DU: 94%, PR: 90%). Conversely, open-loop simulations in which the structure of the neural data was scrambled (black) confirmed that, despite its adaptation capabilities, the RLBMI decoder needed real neural states to perform above chance (50%) levels.

The RLBMI system was very stable over different closed loop sessions, robustly finding an effective control policy regardless of the parameter weights’ initial conditions. Figure 4 shows that during the closed loop robot control experiments, the RLBMI controller selected the correct target in approximately 90% of the trials (blue bar: mean +/− standard deviation; DU: 93%, 5 sessions; PR: 89%, 4 sessions, significantly above chance (0.5) for both monkeys, p<.001, one sided t-test). Similarly, Figure 4 (red bars) shows that the open-loop initial condition Monte Carlo simulations (see **Materials and Methods**) yielded similar accuracy as the closed loop experiments, confirming that the system converged to an effective control state from a wide range of initial conditions (DU: 1000 simulations, PR: 700, significantly above chance (0.5) for both monkeys, p<.001, one sided t-test). The accuracy results in Figure 4 correspond to trials 6–30 since the first 5 trials were classified as an initial adaptation period and the monkeys typically became satiated with food rewards and ceased interacting with the task (e.g. went to sleep, began fidgeting in the chair, otherwise ignore the robot) after 30 to 50 trials.

A surrogate data test was used to confirm that the RLBMI decoder was using the monkeys’ brain activity to control the robot arm, and not some other aspect of the experimental design. These tests involved additional open-loop simulations in which the order of the different trial types recorded during the real-time experiments was preserved while the order of the recorded motor cortex neural data was randomly reshuffled, thus destroying any consistent neural representations associated with the desired robot movements. Despite the decoder’s adaptation capabilities, Figure 4 (black bars) shows that the RLBMI system was not able to perform above chance levels under these conditions (DU: 1000 simulations, PR: 700, p∼1, one sided t-test), demonstrating that the RLBMI was unable to accomplish the task without the direct connection between the motor cortex command signals and the desired robot actions that had been recorded during the real-time experiments.

### RLBMI Stability during Input Space Perturbations: Loss or Gain of Neuron Recordings

**Figure 5:**
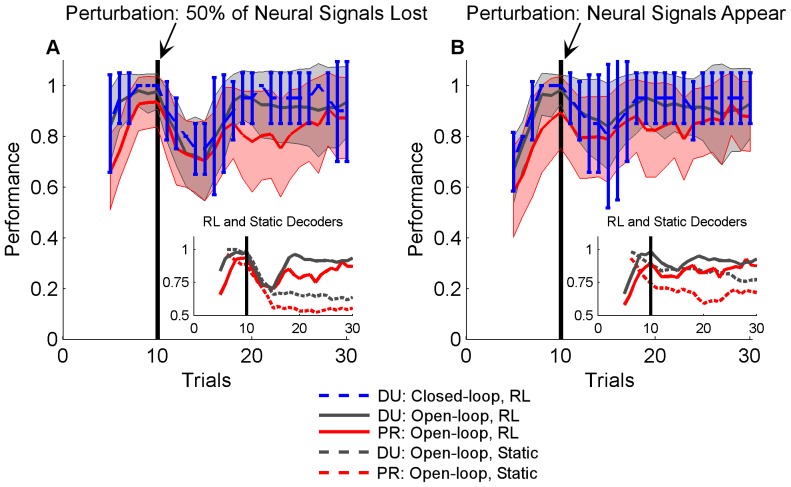
The RLBMI quickly adapted to perturbations to the neural input space. These perturbations included both the loss of 50% of the neural inputs (A), as well as when the number of neural signals detected by the neural recording system doubled (B). (A&B) show the RLBMI performance accuracy within a five-trial sliding window (mean +/− standard deviation). Both closed loop tests (DU: blue dashed line and error bars, 4 sessions) and offline open-loop simulations (DU: gray line and panel, 1000 sims; PR: red line and panel, 700 sims) were used to evaluate the RLBMI response to input perturbations. (A) gives the results of 50% input loss perturbations. In both closed loop experiments and open-loop simulations, the RLBMI had already adapted and achieved high performance by the 10^th^ trial. Following the 10^th^ trial (vertical black bar), 50% of the neural inputs were abruptly lost, with RLBMI readapting to the loss within 5 trials. (B) shows that when the recording electrodes detected new neurons, the RLBMI adaptation allowed the new information to be incorporated into the BMI without the emergence of new firing patterns degrading performance. In these perturbation tests, a random 50% of the available neural signals were artificially silenced prior to the 10^th^ trial (vertical black bar). The sudden appearance of new input information caused only a small performance drop, with the RLBMI again readapting to the perturbation within 5 trials. The inset panels in both (A) and (B) contrast the averaged results of the RLBMI open loop simulations (solid lines, DU: gray, PR: red) with the simulation performance of a nonadaptive neural decoder (dashed lines, a Wiener classifier created using the first five trials of each simulation). In contrast to the RLBMI, the nonadaptive decoder showed a permanent performance drop following perturbations in which neural signals were lost, as well as in the tests in which new signals appeared.

The RLBMI quickly adapted to compensate for large perturbations to the neural input space (see **Materials and Methods**). Figure 5 gives the accuracy (mean and standard deviation) of the RLBMI decoder within a 5-trial sliding window across the trial sequence of both closed-loop BMI experiments (DU: blue dashed line and error bars, 4 sessions) and open-loop simulations (DU: gray line and panel, 1000 simulations; PR: red line and panel, 700 simulations). Figure 5A shows the RLBMI performance when a random 50% of the inputs were lost following trial 10 (vertical black bar). By trial 10 the RLBMI had already achieved stable control of the robot, and it had readapted to the perturbation within 5 trials, restoring effective control of the robot to the monkey. The inset panel in Figure 5A contrasts the mean results of the RLBMI simulations (solid lines) against simulations in which a static neural decoder (dashed lines, specifically a Wiener classifier) was used to generate the robot action commands. The Wiener classifier initially performed quite well, but the input perturbation caused a permanent loss of performance. Figure 5B shows that the RLBMI system effectively incorporate newly ‘found’ neural signals into its input space. This input perturbation again occurred following the 10^th^ trial (vertical black bar), prior to that point a random 50% of the RLBMI inputs had had their firing rate information set to zero. In both the closed-loop BMI experiments and open-loop simulations the system again had adapted to the input perturbation within 5 trials. By comparison, a static decoder (Wiener classifier) was not only unable to take advantage of the newly available neural information, but in fact showed a performance drop following the input perturbation (Figure 5B inset panel, RLBMI: solid lines, static Wiener: dashed lines).

**Figure 6:**
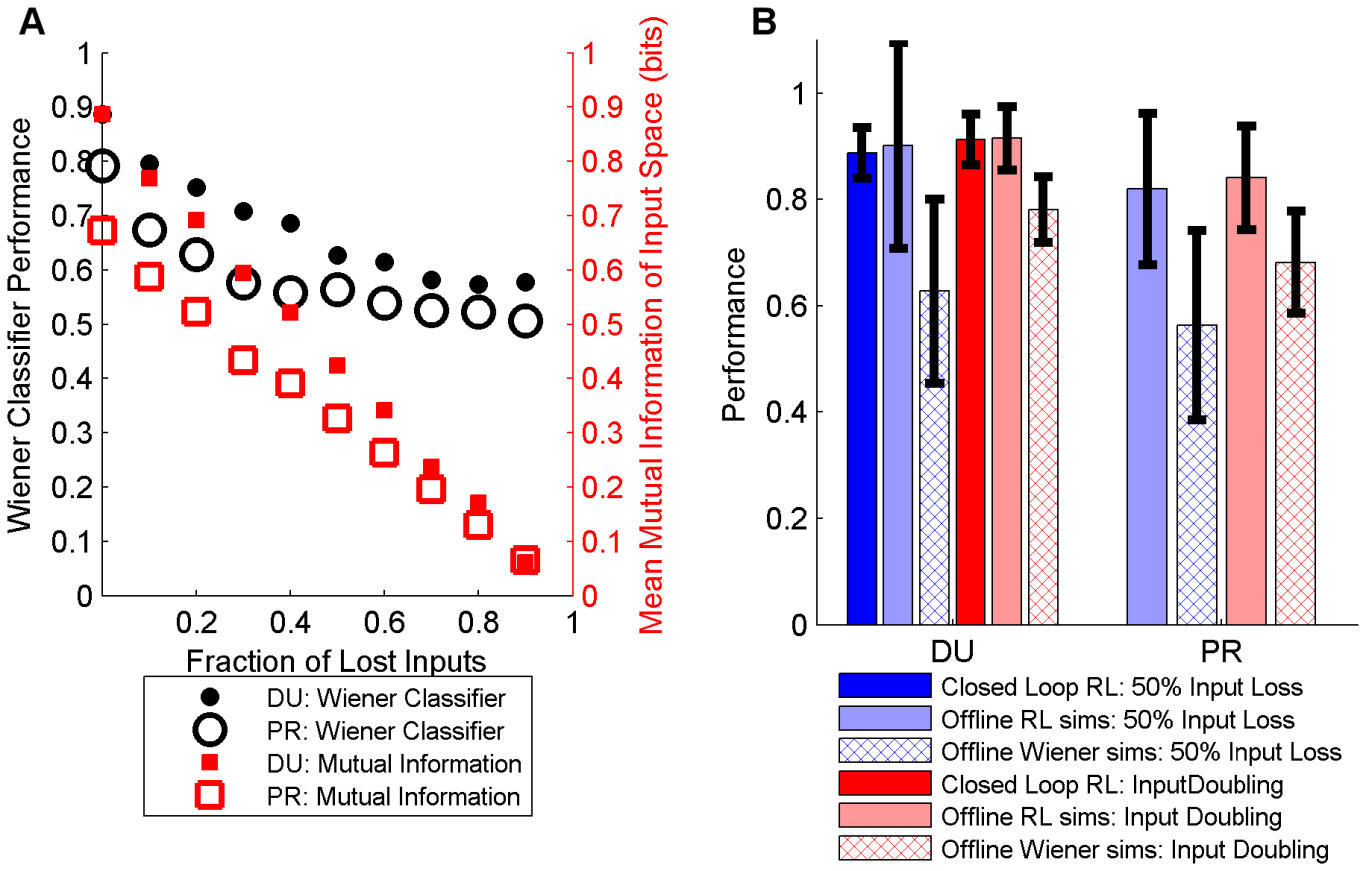
The input perturbations caused significant performance drops without adaptation. (A) displays the effect of different fractions of neural signals being lost on the performance of a nonadaptive neural decoder (Wiener classifier), relative to the average information available from the neural inputs (DU: 1000 simulations; PR: 700 simulations). The average mutual information (equation 5) between the neural signals and the two-target robot task (red boxes; DU: solid, PR: hollow) reflects the magnitude of the input perturbation caused by varying numbers of random neural signals being lost. Losing 50% of the inputs unsurprisingly resulted in a large input shift, with about half the available information similarly being lost by that point for each monkey. It is unsurprising that the cross-validation performance of a nonadaptive neural decoder (black circles; DU: solid, PR: hollow) that had been created prior to the perturbation (Figure 5) thus similarly approached chance performance for such large input losses (performance was quantified as classification accuracy for trials 11 to 30 with the perturbation occurring following trial 10). (B) shows how the RLBMI adapted (Figure 5) to large input perturbations (50% loss of neural signals and doubling of neural signals) during both closed loop experiments (signals lost: dark blue; new signals appear: dark red; 4 experiments) and the offline simulations (signals lost: light blue; new signals appear: light red; DU: 1000 simulations; PR: 700 simulations), resulting in higher performance than the nonadaptive Wiener classifier (hatched boxes, 1sided t-test, p<<.001).

Both the losses of 50% of the recorded inputs and the abrupt appearance of new information amongst half the recordings represent significant shifts to the RLBMI input space. In Figure 6A, we contrast the change in the available information between the neural signals (equation 5) with losses of varying quantities of neural signals (red boxes; DU: solid, PR: hollow). By the time 50% of the inputs have been lost, over half of the information had been lost as well. Abrupt input shifts of this magnitude would be extremely difficult for any static neural decoder to overcome. It is thus not unexpected that the static Wiener classifier (black circles; DU: solid, PR: hollow) nears chance performance by this point, any decoder that did not adapt to the change would show similar performance drops. Figure 6B contrasts the average performance (trials 11 to 30) of the RLBMI following perturbations during both closed loop experiments (neural signals lost: dark blue; new neural signals appearing: dark red) and open loop simulations (neural signals lost: light blue; new neural signals appearing: light red) against the performance of the static Wiener classifier (hatched bars; neural signals lost: blue; new neural signals appearing: red). The RLBMI performance was significantly higher than the nonadaptive Wiener classifier (1sided t-test, p<<.001, DU: 1000 simulations; PR: 700 simulations).

### RLBMI Stability Over Long Time Periods and Despite Input Perturbations

**Figure 7:**
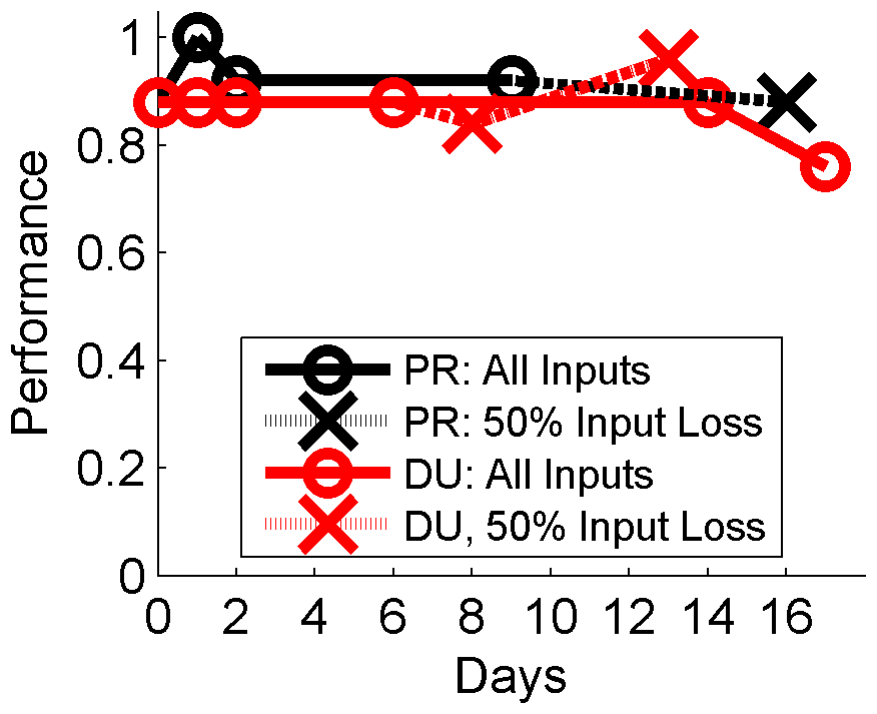
The RLBMI consistently maintained performance across long time periods. The RLBMI was applied in a contiguous fashion across closed loop experimental sessions spanning up to two weeks, and accurately controlled the robot across the sessions (performance defined as accuracy of robot movements during the first 25 trials of each session; O: solid lines). During the first session, the system was initialized with random parameters, and during each subsequent session the system was initialized using parameter weights it had learned previously. This approximates deploying the RLBMI across long time periods since it never has the opportunity to reset the weights and start over, but rather must maintain performance by working with a single continuous progression of parameter weight adaptations. Additionally, despite working with the same sequence of weights for multiple days, the RLBMI was still able to quickly adapt when necessary. A mechanical connector failure caused a loss of 50% of the inputs for PR between day 9 and 16 (X: black dashed line), but the RLBMI adapted quickly and only a small performance drop resulted. This input loss was simulated in two sessions with DU (X: red dashed line), and the system again adapted and maintained performance. Notably, the RLBMI performance during those perturbation sessions was similar or better than in two final DU tests in which no input loss was simulated (in the day 14 session the parameter weights were reset to those learned on day 6).

The RLBMI maintained high performance when applied in a contiguous fashion across experimental sessions spanning up to 17 days, Figure 7. The decoder weights started from random initial conditions during the first session, and during subsequent sessions the system was initialized from weights learned in the previous session (from the 25^th^ trial), and was then allowed to adapt as usual (equations 3 and 4) without any new initializations or interventions by the experimenters, this was done to approximate use of the BMI over long time periods. The solid lines in Figure 5 give the accuracy of the system during the first 25 trials (mean: DU: 86%; PR: 93%) of each session when the inputs were consistent. For monkey PR, half of the neural input signals were lost between day 9 and 16 (dashed line). However, the system was able to quickly adapt and this loss resulted in only a slight dip in performance (4%), despite the fact the RLBMI had been adapting its parameters for several days to utilize the original set of inputs. Likewise, the RLBMI controller maintained performance during two DU sessions (day 8 and 13, dashed line) in which a similar input loss was simulated (see **Materials and Methods**). In fact, performance during those sessions was similar or better to DU tests that continued to use all the available neural signals (days 14 and 17).

## Discussion

### Potential Benefits of using Reinforcement Learning Algorithms for BMI Controllers

Adaptive and interactive algorithms based on reinforcement learning offer several significant advantages as BMI controllers over supervised learning decoders. First, they do not require an explicit set of training data to be initialized, instead being computationally optimized through experience. Second, RL algorithms do not assume stationarity between neural inputs and behavioral outputs, making them less sensitive to failures of recording electrodes, neurons changing their firing patterns due to learning or plasticity, neurons appearing or disappearing from recordings, or other input space perturbations. These attributes are important considerations if BMIs are to be used by humans over long periods for activities of daily living.

### The Reinforcement Learning BMI System does not Require Explicit Training Data

The RLBMI architecture did not require an explicit set of training data to create the robot controller. BMIs that use supervised learning methods require neural data that can be related to specific BMI behavioral outputs (i.e. the training data) to calibrate the BMI. In many BMI experiments that have used healthy nonhuman primate subjects, the training data outputs were specific arm movements that accomplished the same task for which the BMI would later be used [7]–[11], [28], [50]–[52]. While those methods were effective, gaining access to this type of training data is problematic when considering paralyzed BMI users. Other studies have found that carefully structured paradigms that involve a BMI user first observing (or mentally imagining) desired BMI outputs, followed by a process of refinements that gradually turn full BMI control of the system over to the user, can provide training data without physical movements [1], [6], [22]. While these methods were again effective, they require carefully structured BMI paradigms so that assumed BMI outputs can be used for the calibration. The RLBMI system shown here avoids such issues. Calculating the parameters of the RLBMI architecture never requires relating neural states to known (or inferred) system outputs, but rather the system starts controlling the robot with random parameters which are then gradually adapted given feedback of current performance. Thus, the robot made random movements when the system was initialized (as can be seen in Figure 3A), but the RLBMI was able to quickly (typically within 2–4 trials) adapt the parameters to give the monkeys accurate control (∼90%) of the robot arm. This adaption only required a simple binary feedback. Importantly, the same RLBMI architecture utilized here can be directly applied to tasks that involve more than two action decisions, while still using the exact same weight update equations. This means that the system can be readily extended to more sophisticated BMI tasks while still only requiring the same type of binary training feedback [34], this opens numerous opportunities for RLBMI deployment with paralyzed users. Finally, not relying on explicit training data helped make the RLBMI system stable over long time periods (∼2 weeks), since the architecture continually refined its parameters based on the user’s performance to maintain control of the robot arm, as shown in Figure 7.

### The Reinforcement Learning BMI System Remained Stable Despite Perturbations to the Neural Input Space

It is important that changes in a BMI’s neural input space do not diminish the user’s control, especially when considering longer time periods where such shifts are inevitable [53]–[57]. For example, losses and gains of neurons are very common with electrophysiology recordings using chronically implanted microelectrode arrays: electrodes fail entirely, small relative motions between the brain and the electrodes cause neurons to appear and disappear, and even the longest lasting recording arrays show gradual losses of neurons over time from either tissue encapsulation of the electrodes or from the gradual degradation of the electrode material [58], [59]. While some changes in neural input behavior can be beneficial, such as neurons gradually adopting new firing patterns to provide a BMI user greater control of the system [8], [10], [17], [18], [46]–[48], large and/or sudden changes in neuron firing patterns will almost always reduce a BMI user’s control if the system cannot compensate, as can be seen in performance drop of the static Wiener decoder in Figures 5 and 6.

While input losses may be an obvious adverse perturbation to BMI systems (as shown in Figure 6), the appearance of new neurons is also a significant input perturbation: when the representations of new neurons overlap with neurons that were already being used as BMI inputs, this causes the previous inputs to appear to have acquired new firing patterns, thus perturbing the BMI’s input space. Such appearances could be a particular issue in BMI systems that rely on action potential threshold crossings on a per electrode basis to detect input activity [21], [52], [60]. Finally, BMIs that cannot take advantage of new sources of information lose the opportunity to compensate for losses of other neurons.

Currently, most BMI experiments avoid the issue of large changes in input neurons on BMI performance since the experimenters reinitialize the systems on, at least, a daily basis [1]–[3], [6], [21]–[23]. However, it is important for practical BMI systems to have a straightforward method of dealing with neural input space perturbations that are not a burden on the BMI user and do not require such daily recalibrations. The RLBMI controller shown here does not require the intervention of an external technician (such as an engineer or caregiver) to recalibrate the BMI following changes in the input space. Rather, it automatically compensates for input losses, as demonstrated in Figure 5 in which the RLBMI adapted and suffered only a transient drop in performance despite neural signals disappearing from the input space. Similarly, Figures 5 and 6 show how the RLBMI automatically incorporated newly available neural information into the input space. Figure 5 shows that the RLBMI did display greater variation in performance following the addition of new inputs compared to its performance following input losses. This may reflect the variability to which the RLBMI algorithm had learned to ignore initially silent channels, combined with the variation in the magnitude of the firing activity of the neural signals once they were ‘found’. In situations in which the algorithm had set the silent channel parameter weights very close to zero, or in which the activity of the new channels was relatively low, the addition of the new neural signals would have had little impact on performance until the RLBMI controller reweighted the perturbed inputs appropriately to effectively use them. Conversely, during the input loss tests there would be a higher probability that dropped inputs had had significant weight parameters previously attached to their activity, resulting in a more obvious impact on overall performance when those neural signals were lost. Finally, since the RLBMI constantly revises which neural signals, and by extension which electrodes, to use and which to ignore as BMI inputs, engineers or caregivers initializing RLBMI systems would not to spend time evaluating which electrodes or neurons should be used as BMI inputs.

The RLBMI architecture is intended to balance the adaptive nature of the decoder with the brain’s learning processes. Understanding the intricacies of these dynamics will be an important focus of future work for the study of brain function and BMI development. Numerous studies have shown that neurons can adapt to better control BMIs [8], [10], [17], [18], [46]–[48]. RL adaptation is designed so that it does not confound these natural learning processes. RL adaption occurs primarily when natural neuron adaptation is insufficient, such as during initialization of the BMI system or in response to large input space perturbations. Figures 3 and 5 show the current RLBMI architecture offers smooth adaptation and stable control under both such conditions. In the current experiments, the speed and accuracy of the RLBMI balanced any adaptation by the recorded motor cortex neurons themselves. Further research that combines studies of natural neuron learning capabilities with more complicated BMI tasks will be necessary though to develop RLBMI architectures that can provide mutual optimal adaptation of both the brain and the neural decoder, and thus offer highly effective and robust BMI controllers.

### Obtaining and using Feedback for Reinforcement Learning BMI Adaptation

The ability of the RLBMI system to appropriately adapt itself depends on the system receiving useful feedback regarding its current performance. Thus both how accurate the critic feedback is and how often it is available directly impacts the RLBMI’s performance. The current experimental setup assumed an ideal case in which completely accurate feedback was available immediately following each robot action. While such a situation is unlikely in everyday life, it is not essential for RL that feedback always be available and/or correct, and there are many potential methods by which feedback information can be obtained.

The RLBMI architecture presented here does not intrinsically assume perpetually available feedback, but rather only needs feedback when necessary and/or convenient. If no feedback information is available, then the update equations are simply not implemented and the current system parameters remain unchanged. Since feedback information does not depend on any particular preprogrammed training paradigm, but rather simply involves the user contributing good/bad information during whatever task for which they are currently using the BMI, this makes the system straightforward to update by the user whenever is convenient and they feel the RLBMI performance has degraded. Finally, other RL algorithms are designed specifically to take advantage of only infrequently available feedback by relating it to multiple earlier actions that were taken by the system and which ultimately lead to the feedback [61].

**Figure 8:**
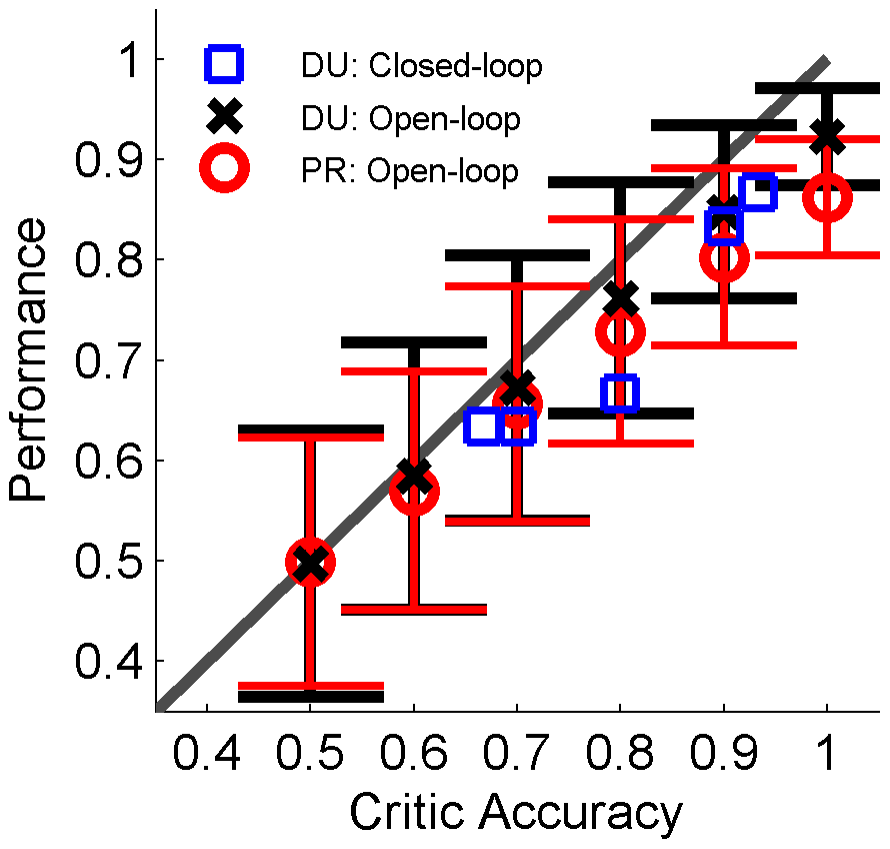
Accuracy of critic feedback impacts RLBMI performance. Shown is the accuracy of the RLBMI system (trials 1∶30) during closed loop sessions (DU: blue squares, 5 sessions) and during open loop simulations (mean +/− standard deviation; DU: black X, 1000 simulations; PR: red O, 700 simulations) when the accuracy of the critic feedback was varied (0.5 to 1.0). Gray line gives a 1∶1 relationship. The RLBMI performance was directly impacted by the critic’s accuracy. This suggests that choosing the source of critic feedback must involve a balance of factors such as: accessibility, accuracy, and frequency of feedback information, with adaptation preferably only being implemented when confidence in the feedback is high.

When considering possible sources of feedback information, it is important to consider how the critic accuracy impacts on the RLBMI’s overall performance. We thus ran a several closed loop experiments and offline simulations in which we tested how well the RLBMI algorithm was able to classify trials from the closed loop BMI experiments when the accuracy of the critic feedback varied. Figure 8 shows how the RLBMI performance can be limited by the accuracy of the feedback. Thus for the current RLBMI architecture it may be better to only use feedback information when the confidence in its accuracy is high, even if that means feedback is obtained less frequently.

There are a wide variety of potential options for the RLBMI user to provide critic feedback to the system, including using reward or error information encoded in the brain itself. While assuming that ideal feedback is available following each action may not be practical for real BMI systems, the fact that the necessary training feedback is just a binary ‘good/bad’ signal (even when the system is expanded to include more than two output actions) that only needs to be provided when the user feels the BMI performance needs to be updated, leaves many options for how even a user suffering from extreme paralysis could learn to provide critic feedback. For example, the user could use a breath puff system, vocal cues, or any sort of small residual movement or EMG signal that can be reliably evoked. Furthermore, error related signals characteristic to EEG, ECoG, or other recording methods could be employed as well [32], [62]–[67]. An exciting option that would place the smallest burden on the BMI user would be to automatically decode feedback information regarding the BMI’s performance directly from the brain itself, perhaps from learning or reward centers such as the nucleus accumbens, anterior cingulate cortex, prefrontal cortex etc. [68]. More research will be necessary to investigate potential sources of feedback information that the BMI user could easily provide, as well as how to best and how frequently to use that feedback for effective adaptation by the RLBMI architecture. Giving the BMI user access to a straightforward method of providing feedback will enable them to use the BMI system effectively over long periods of time without outside interventions by engineers or caregivers despite inevitable changes to the inputs. This will greatly increase the practicality of BMI systems by increasing user independence.

## Conclusions

These experiments highlight several of the advantages offered by reinforcement learning algorithms when used as BMI controllers: the system can learn to control a device without needing an explicit set of training data, the system can robustly adapt and maintain control despite large perturbations in the input signal space, and control can be maintained across long time periods. Two marmoset monkeys used an actor-critic Reinforcement Learning BMI to control a robot arm during a reaching task. The RLBMI system was initialized using random initial conditions, and then used a binary training feedback signal to learn how to accurately map the monkeys’ neural states to robot actions. The system achieved 90% successful control of the arm after only 2–4 trials, and could maintain control of the arm across sessions spanning days to weeks. Furthermore, because the RLBMI continuously adapted its parameters, it was quickly (within 4 to 5 trials) able to regain control of the robot when half the BMI input neural signals were abruptly lost, or when half the neural signals suddenly acquired new activity patterns. The advantages of the adaptive algorithm illustrated here offer a means for future BMI systems to control more complicated systems with a reduced need for recalibration or other outside inventions by external agents such as engineers or caregivers, which would greatly increase independence to the BMI user.

## Supporting Information

Movie S1 RLBMI control of the robot arm during a 50% loss of inputs. This movie was recorded during a session in which the monkey used the RLBMI to move the robot between two targets. Shown are trials 1–4 and 11–16. The RLBMI learned an effective mapping of the neuronal inputs to the desired robot actions within the first few trials. After the tenth trial, the system was perturbed by dropping 50% of the neuronal inputs, forcing the RLBMI to automatically adapt in order to restore effective control of the robot to the monkey.
(WMV)
